# STAT3 phosphorylation in central leptin resistance

**DOI:** 10.1186/s12986-021-00569-w

**Published:** 2021-04-13

**Authors:** Huimin Liu, Tianxin Du, Chen Li, Guoqing Yang

**Affiliations:** College of Life Science, Henan Agricultural University, 95 Wen Hua Road, Zhengzhou, 450002 China

**Keywords:** Leptin resistance, STAT3 phosphorylation, Hypothalamus, DIO, Mechanism

## Abstract

Mechanism exploitation of energy homeostasis is urgently required because of the worldwide prevailing of obesity-related metabolic disorders in human being. Although it is well known that leptin plays a central role in regulating energy balance by suppressing food intake and promoting energy expenditure, the existence of leptin resistance in majority of obese individuals hampers the utilization of leptin therapy against these disorders. However, the mechanism of leptin resistance is largely unknown in spite of the globally enormous endeavors. Current theories to interpret leptin resistance include the impairment of leptin transport, attenuation of leptin signaling, chronic inflammation, ER tress, deficiency of autophagy, as well as leptin itself. Leptin-activated leptin receptor (LepRb) signals in hypothalamus via several pathways, in which JAK2-STAT3 pathway, the most extensively investigated one, is considered to mediate the major action of leptin in energy regulation. Upon leptin stimulation the phosphorylation of STAT3 is one of the key events in JAK2-STAT3 pathway, followed by the dimerization and nuclear translocation of this molecule. Phosphorylated STAT3 (p-STAT3), as a transcription factor, binds to and regulates its target gene such as POMC gene, playing the physiological function of leptin. Regarding POMC gene in hypothalamus however little is known about the detail of its interaction with STAT3. Moreover the status of p-STAT3 and its significance in hypothalamus of DIO mice needs to be well elucidated. This review comprehends literatures on leptin and leptin resistance and especially discusses what STAT3 phosphorylation would contribute to central leptin resistance.

## Introduction

The greatly increased prevalence of type 2 diabetes and some metabolic diseases closely related with the rapidly growing obesity epidemic around the world, which due to an energy imbalance caused by unhealthy lifestyles and nutrient overload [1]. A better understanding of the mechanism underlying obesity is crucial in the battle against obesity. Increasing reports demonstrated that a vital risk factor for obesity is leptin resistance. However, the mechanisms that lead to leptin resistance are largely remains elusive. Emerging evidence provided insight into the role of signal transducer and activator of transcription 3 (STAT3) in energy metabolism. Therefore, elucidating the functions of STAT3 in energy metabolism and leptin resistance is necessary. In this review, the elaborate picture of STAT3 in leptin resistance is presented, which should help to facilitate the potential of STAT3 as a future therapeutic target.

## Leptin

### Leptin discovery

A complex control system of energy metabolism in vertebrates, especially mammalian species, has been established during the long period of evolution. The ability to store large quantities of energy against the food deprivation is on one hand crucial for survival, but on the other hand potential for the occurrence of metabolic problems in human beings because of food over-consumption and/or lack of physical movement in modern life. Worldwide metabolic disorders like obesity, diabetes and cardiovascular diseases are on the rise, making endeavors in metabolism study an urgent task for scientists. A milestone in metabolism field is the discovery in 1994 made by Friedman and his colleagues, which convincingly showed that leptin are essential in regulating food intake and energy expenditure [2].

Leptin is a peptide hormone (16-kDa) encoded by the obese (*ob*) gene. It is mainly produced in the white adipose tissue, and secreted in proportion to fat mass [3, 4]. The discovery of leptin begins with a mouse model in the Jackson laboratory [5]; where the *ob* gene was identified as a key regulator for obesity [2, 6]. Subsequently, the protein product of the *ob* gene was named “leptin” derived from the Greek word leptos, meaning “thin”.

### Leptin function

Leptin is a pleotropic hormone in energy metabolism. Animals without leptin signaling display severe metabolic imbalance. For example, mice with genetic deficiency of leptin (ob/ob) or leptin receptor gene (db/db) suffers from hyperphagia, severe obesity and diabetes, while administration of recombinant leptin from bacteria reverses these disorders in ob/ob, but not in db/db mice, indicating leptin depends on its receptor for displaying functions [7]. The circulating leptin signals the status of body energy stores to the brain and induces the satiety responses through neurotransmmiters. Therefore, as a long-term regulator, leptin suppresses food intake to control the body weight.

Besides, leptin has been shown to promote energy expenditure particularly through its effects on the brown adipose tissue (BAT) thermogenesis via the hypothalamic. While ob/ob mice show low body temperature [8], leptin administration or the artificial activation of leptin receptor in hypothalamus increases metabolic activity of rodents with body weight loss [9]. This observation is consistent with the finding that leptin initiates the thermogenesis of BAT [10]. Hypothalamic administration of leptin elevates the level of blood catecholamine, suggesting that the leptin act on thermogenesis is through the sympathetic nervous system (SNS). However, it is still not clear how leptin regulate the activity of SNS via hypothalamus [11].

## Leptin signaling pathway

### Leptin receptor and its signaling

**Fig. 1:**
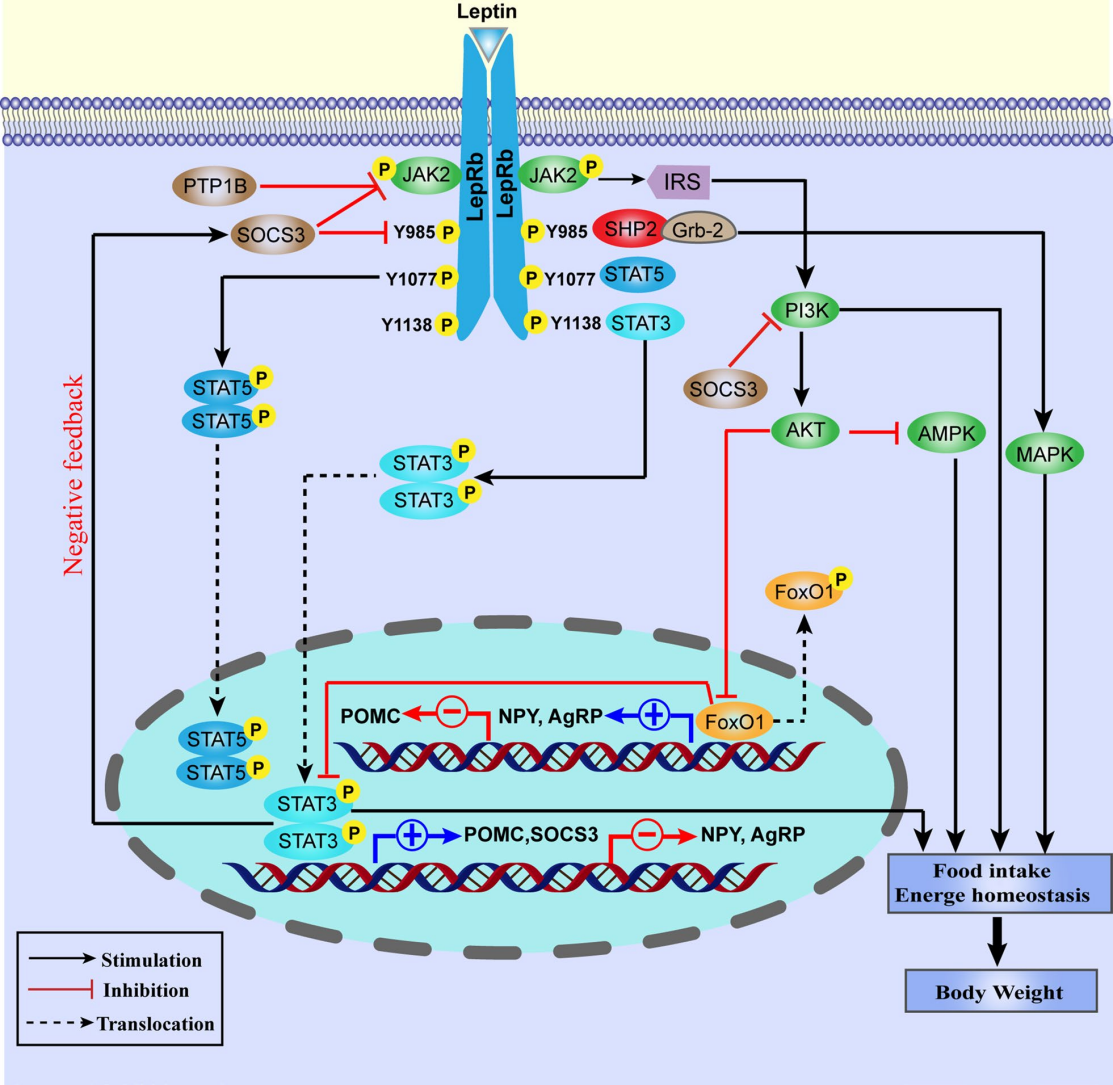
Leptin signaling pathways. Leptin binding to LepRb activates JAK2, thus leading to phosphorylation of LepRb on Tyr985, Tyr1077 and Tyr1138. Phospho-Tyr985, -Tyr1077 and-Tyr1138 bind to downstream molecules and activate the JAK2/STAT3, JAK2/STAT5, PI3K/IRS/AKT, and SHP2/MAPK pathways. Phosphorylated STAT3 dimers translocate to the nucleus and induce the transcription of target genes (POMC, SOCS3). SOCS3 inhibits the JAK2/STAT3 pathway by interacting with phospho-Y985 or JAK2 and acting as a feedback inhibitor of leptin signaling. PTP1B also inhibits leptin signaling through dephosphorylation of JAK2. After JAK2 activation, SHP2 binds to phosphor -Y985 in the LepRb and recruits the adaptor protein Grb2, causing activation of the ERK signaling cascade. Leptin also regulates PI3K signaling through IRS phosphorylation. Leptin has also been shown to stimulate the POMC neurons, while inhibit NPY/AGRP. FoxO1 is an important downstream target of PI3K in the leptin signaling pathway. These pathways act coordinately to regulate food intake, energy balance and body weight

Leptin exerts biological actions by interacting with leptin receptors (LepRs) that is widely distributed in the brain and peripheral tissues [12, 13]. The central nervous system (CNS), especially the hypothalamus, rather than peripheral tissues, is identified to be the main leptin target for the regulation of physiological functions [14]. Multiple splice variants of LepR gene encode at least six different LepRs isoforms, which have an identical extracellular leptin-binding domain at N-terminal but different intracellular domains at the C-terminal [15]. The isoforms are divided into three types: short (LepRa, c, d, and f), long (LepRb), and secreted (LepRe) forms [16] (Fig. 1). Only LepRb contains the domain required for JAK-STAT signaling pathway [17–19]. As expected, db/db mice which harbors mutated lepRb display a phenotype similar to leptin-deficient ob/ob mice, suggesting that LepRb is the most critical in mediating leptin’s actions [14]. Short-form LepRs may be involved in leptin transport and leptin clearance [20, 21], and LepRe is the soluble secreted one [22].

**Fig. 2:**
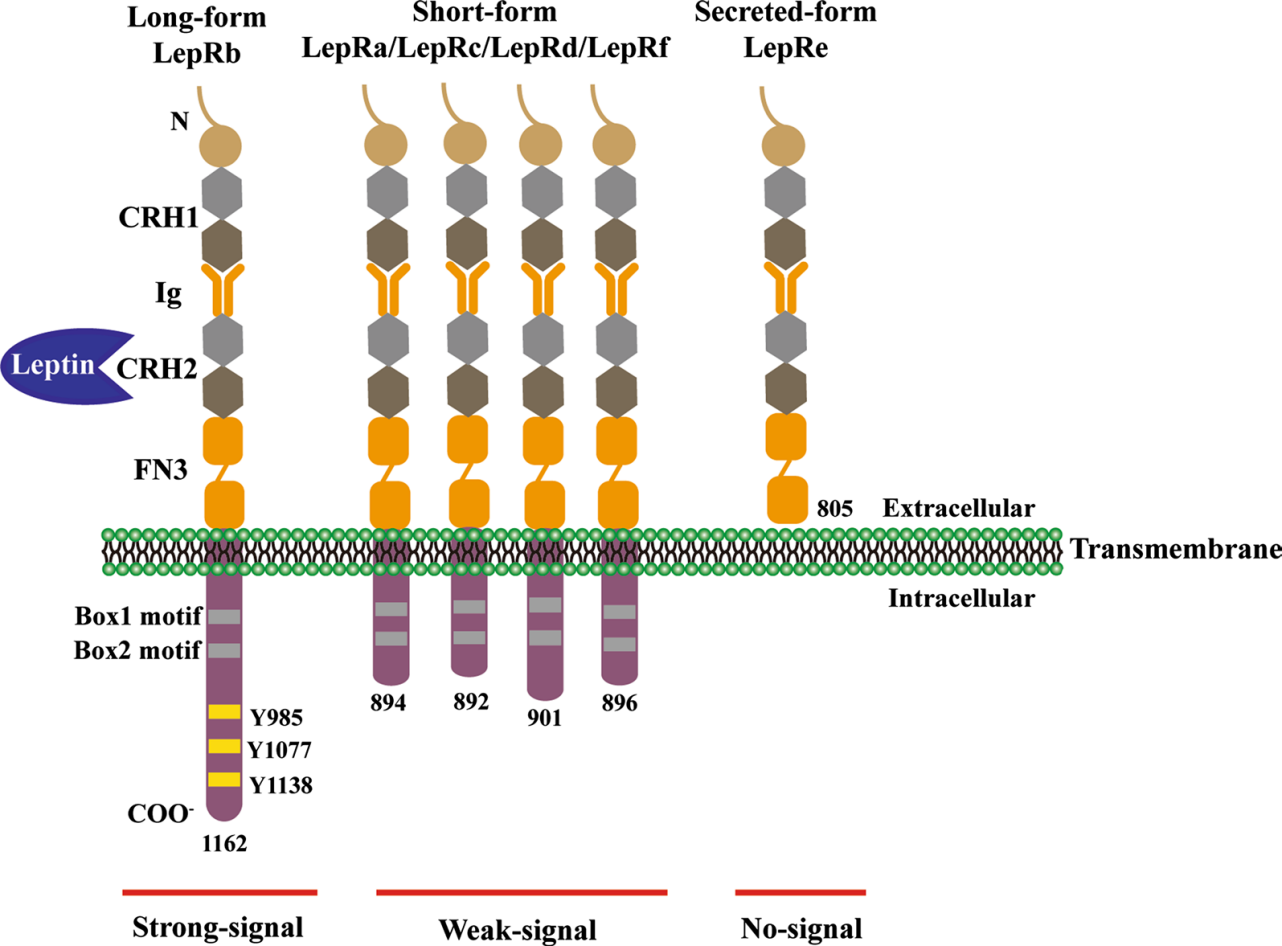
Schematic representation of leptin receptors isoforms. Six different spliced isoforms of the leptin receptors (LepR) have been documented as LepRa–LepRf. All the isoforms share identical extracellular ligand-binding domain formed from the second segment of cytokine receptor homology (CRH) and fibronectin type III domain (FN3). LepRs presents a variable-length cytoplasmic portion (except the soluble receptor LepRe). LepRb, the long form of the receptor, is the only isoform that contains three tyrosine conserved regions (Y985, Y1077, and Y1138) enabling the leptin-induced activation of the JAK-STAT pathway. Box1 and Box2 are involved in JAK association and activation. Ig, immunoglobulin domain; JAK, Janus kinase

Activation of LepR directly or indirectly activates multiple signaling pathways, including Janus tyrosine kinase 2 (JAK2)/STAT3, srchomology-2-containing protein tyrosine phosphatase 2 (SHP2)/growth factor receptor-bound protein 2 (Grb2)/mitogen-activated protein kinase (MAPK), forkhead box O1 (FoxO1), 5′ adenosine monophosphate-activated protein kinase (AMPK) and others. These pathways act synergistically to fully mediate leptin function (Fig. 2). Of these, JAK2/STAT3 pathway is the best characterized. Functional LepRb belongs to the type I family of cytokine receptors without the activity of an internal kinase [23]. Binding of leptin to LepRb initiates a cascade of signaling pathway beginning with the activation of JAK2, which is in turn autophosphorylated. The activated JAKs, especially JAK2 stimulates the phosphorylation of the three residues on the intracellular domain of LepRb (Tyr_985_, Tyr_1077_ and Tyr_1138_). Each of the three phosphorylation sites recruits distinct downstream signaling molecules, and induces a leptin-specific signaling pathway with diverse physiological actions [24].

### JAK2/STAT3 signaling

In response to leptin, JAK2 activated LepRb on Tyr_1138_ and phospho-Tyr_1138_ binds to the Src homology 2 (SH2) domain of STAT3, which is then phosphorylated via LepRb-associated of JAK2 [25, 26]. Phosphorylated STAT3 is translocated from the cytoplasm into the nucleus, where it acts as a transcription factor to regulate the expression of its target genes, including suppressor of cytokine signaling 3 (SOCS3) and neuropeptides [27, 28]. Numerous genetic evidence demonstrates that the JAK2/STAT3 signaling appears to play a major role in energy homeostasis and neuroendocrine function. Furthermore, a specific deletion of Tyr_1138_ in LepRb or STAT3 in LepRb-expressing neurons leads to hyperphagia and obesity similar to the phenotype observed in db/db mice [29], suggesting that Tyr_1138_ in STAT3 is vital for leptin action.

### JAK2/STAT5 signaling

Leptin stimulates LepRb phosphorylation on Tyr1077, and phosphorylated Tyr_1077_ binds to the SH2 domain of STAT5 and makes it phosphorylated and activated by JAK2 [30]. Phospho-Tyr1138 also partially contributes to STAT5 activation [31]. Mice with a STAT5 deletion in the brain causes hyperphagia and obesity, whereas activation of STAT5 in hypothalamic neurons inhibits food intake [32], but less severely than in mice with a STAT3 deletion, suggesting that the JAK2/STAT5 signaling plays a minor role in leptin’s regulation of feeding and body weight [33]. A recent research reveals that ablation of STAT5 in LepRb neurons fails to change energy balance [34].

### SHP2/ERK signaling

Phosphorylation of Tyr_985_ activates signaling related with SH2-containing protein tyrosine phosphatase 2 (SHP2) and mediates the leptin-stimulated activation of the extracellular signal-related kinase (ERK) pathway, which executes the important functions in thermogenesis and anti-obesity of leptin [35, 36]. SHP2 may also downregulate JAK2/STAT3 signaling under some conditions [37]. Neuron-specific deletion of the SHP2 results in obesity and leptin resistance in mice, suggesting that the SHP2 pathway is also important in mediating leptin’s anti-obesity action [38]. Phospho-Tyr985 also binds to the SH2 domain of SOCS3, and SOCS3 in turn inhibits the activation of the LepRb/JAK2 pathways [39]. One study reported that ablation of Tyr985 phosphorylation by a replacement of Tyr985 with Phe promotes diet-induced leptin resistance and obesity [40]. Surprisingly, another study reported that elimination of Tyr985 phosphorylation protects against diet induced obesity in female mice [41]. It is likely that the levels of intracellular SOCS3 may contribute to the outcome of phosphorylation of Tyr985.

## STAT3 in JAK2/STAT3 signaling pathway of leptin

### Structure and function of STAT3

The STATs, a family of transcription factors (STAT1, 2, 3, 4, 5a, 5b, and 6) [42], exert vital functions in signal transduction and transcriptional regulation [43–45]. STAT3, a member of STATs, was first identified in 1994 and is highly conserved [46]. Similar to other STAT proteins, STAT3 is composed of the 6 domains: N-terminal dimerization domain (ND), coiled-coil domain (CCD) for protein–protein interactions, central DNA-binding domain (DBD), linker domain (LD) that affects DNA-binding stability, SH2 domain essential for STAT3 activation, and a C-terminal transcriptional activation domain (TAD) with a conserved tyrosine residue at 705 (Tyr705) and a serine phosphorylation site at 727 (Ser727) [47].

**Fig. 3:**
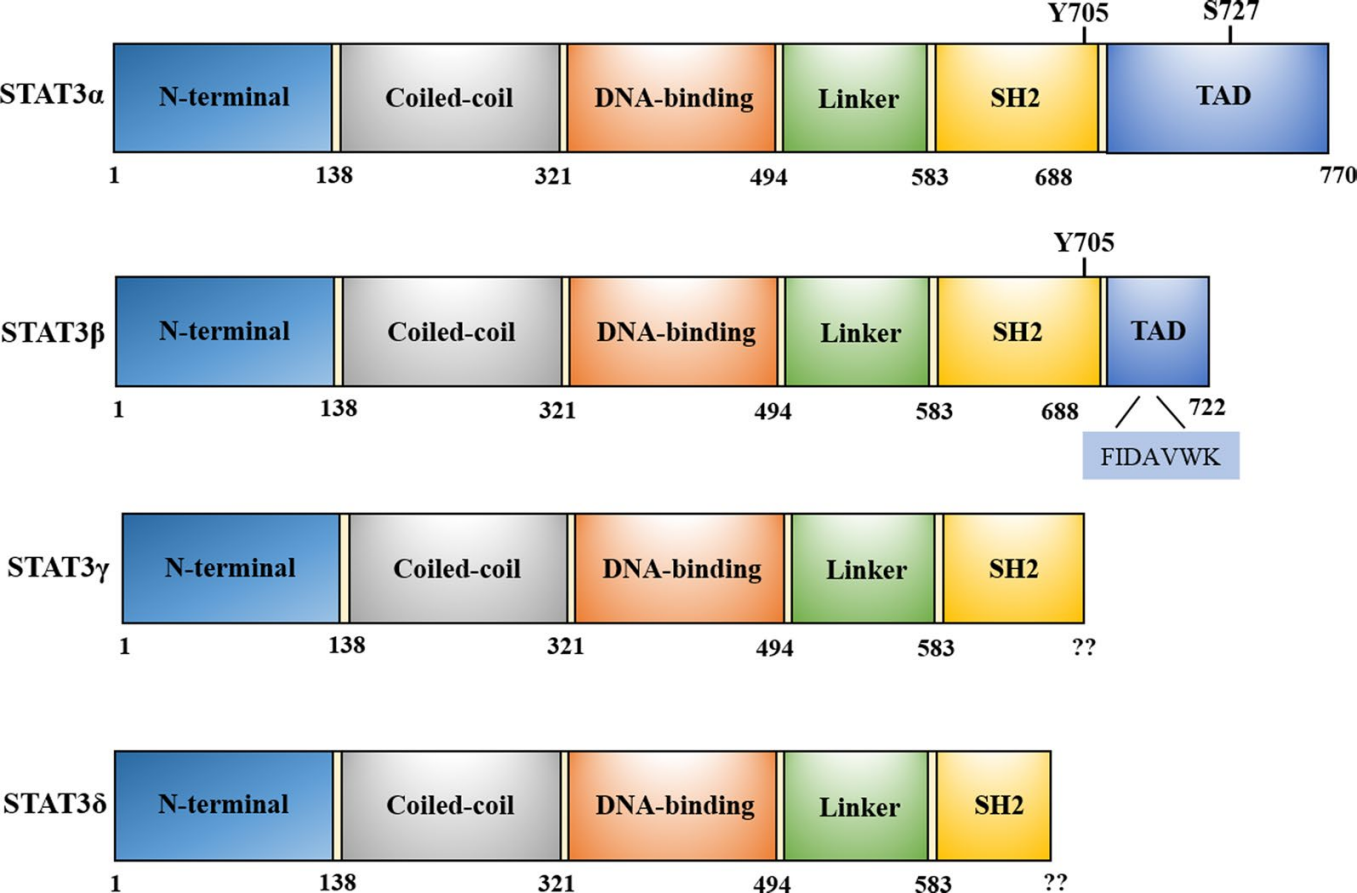
Schematic representation of STAT3 isoforms domains. STAT3 structure is composed by a helical N-terminus domain (NTD); a coiled-coil domain (CCD); a central DNA-binding domain (DBD); a linker domain (LD); an Src homology 2 (SH2) domain; and a C-terminal transactivation domain (TAD) with a conserved tyrosine residue at 705 (Y705) and a serine phosphorylation site at 727(S727)

Additionally, STAT3 has been identified as having four distinct subtypes: STAT3α (92 kDa), STAT3β (83 kDa), STAT3γ (72 kDa) and STAT3δ (64 kDa), which are regarded a pivotal determinant of STAT3 functional heterogeneity [48–50]. STAT3β, an alternative splicing transcript of full-length STAT3α, replaces the C-terminal 55 amino acids with 7 specific amino acid tail (Fig. 3), leading to greater DNA binding activity [51]. Notably, it has been recently proved that STAT3β has distinct regulatory and transcriptional functions, which might have a suppressive effect on STAT3 [51–53]. STAT3α exert its functions in modulation of cellular responses to IL-6 and IL-10 in macrophages [54]. STAT3γ and STAT3δ, the other two STAT3 isoforms, are derived from the proteolytic processing of STAT3, which were mainly activated in different stages of granulocytic differentiation [48] (Table 1).

**Table 1:** STAT3 isoforms

STAT3 isoform	Molecular weight (kDa)	Structure	Function	References
STAT3α	92	Full length	Typical STAT3 function	[47]
STAT3β	83	C-terminal truncated form (missing Ser727)	Greater DNA binding activity; Distinct regulatory and transcriptional functions; Suppressive effect on STAT3	[51]
STAT3γ	72	C-terminal truncated form (missing Ser727and Tyr705)	Activated in the terminal stage of granulocytic differentiation	[48]
STAT3δ	64	C-terminal truncated form (missing Ser727 and Tyr705)	Activated in the early stage of granulocytic differentiation	[48]

### STAT3 targets in neurons

The hypothalamus is considered a key organ in the regulation of food intake. Leptin primarily acts on the hypothalamic arcuate nucleus (ARC) neurons producing orexigenic neuropeptide Tyr (NPY)/*Agouti*-related protein (AgRP) and anorexigenic proopiomelanocortin (POMC)/amphetamine-regulated transcript (CART) [55, 56]. Leptin promotes POMC gene expression and activates POMC neurons [57–59], while decreases AgRP gene expression and inhibits AgRP neurons [60, 61]. Consistently, *ob/ob* and *db/db* mice display elevated levels of AgRP mRNA and reduced levels of POMC mRNA [62, 63]. Ablation of AgRP neurons and decrease in AgRP gene expression lead to lean mice [64, 65]. Conversely, deletion of POMC neurons and loss of POMC-derived transmitters result in obesity [65, 66]. This underlines the importance of leptin-stimulated POMC and AgRP neuron regulation in the control of food intake and energy homeostasis [67]. In addition, leptin has also been shown to stimulate the expression of the transcription factor steroidogenic factor-1 (SF-1) neurons in the ventromedial hypothalamus (VMH), leading to inhibition of feeding [68]. Like hypothalamic neurons, the nucleus tractus solitaries (NTS) POMC neurons showed the STAT3 activation in response to exogenous leptin [69].

Leptin-stimulated STAT3 dimer binds to the POMC and AgRP promoters in the nucleus, which stimulates the expression of POMC and suppresses that of AgRP, thereby decreasing food intake and increasing energy expenditure. There are two STAT3 binding sites in POMC promoter, the distal (− 361 to − 353) and the proximal (− 76 to − 68). With regard to POMC gene regulation, it has been reported that the POMC promoter contains forkhead DNA-binding sites adjacent to distal site, and FoxO1 competes with STAT3 for binding to neighboring DNA sequences in the POMC promoter, thereby inhibiting leptin-mediated POMC expression [70]. However, we [71] and other [72] demonstrated in the cultured cells that deletion of the distal STAT3-binding site had little effect, but the removal of the proximal one dramatically abrupt leptin regulation of POMC promoter activity.

Numerous mouse models with neuronal ablation of STAT3 have been proved essential roles of STAT3 in energy homeostasis regulation. Neural-specific disruption of STAT3 (STAT3^N−/−^) in mice [73] and mice with mutated leptin receptors that do not bind STAT3 [29] are hyperphagic, and obese with decreased energy expenditure, and show raised mRNA levels of AgRP. Interestingly, it has been reported that leptin-activated STAT3 is not required for the development of leptin response in the AgRP neurons [74, 75]. Notably, mice deficient of STAT3 in AgRP neurons appears to have no change in the mRNA levels of AgRP [76]. The deletion of STAT3 in the CNS leads to severe obesity with accompanying decreased POMC expression [73], whereas STAT3 inactivation in POMC neurons causes slight obesity and diminished POMC expression, which suggest STAT3 as a transcriptional activator of POMC expression [77]. Similarly, the disruption of STAT3 in POMC or/and AgRP neurons provokes only modest effects on food intake and body weight [77, 78]. Therefore, although LepRb-expressing POMC and AgRP neurons are both crucial for leptin action, to a large extent, LepRb in these neurons is dispensable [79].

### STAT3 activation

In unstimulated cells, STAT3 is inactive and presents as monomer in the cytoplasmic [54, 80]. Once activated by various types of stimuli, phosphorylated STAT3 translocate into the nucleus to initiate transcription via binding to the promoter sequence of target genes. Moreover, it has been reported that STAT3 nuclear import may be independently of its phosphorylation and mediated by specific import carrier-α3 [81]. Therefore, STAT3 can exert functions by formation of phosphorylated STAT3 (p-STAT3) dimers as well as enhanced amounts of unphosphorylated STAT3 in cytokine-dependent transcription.

Apart from the classical pathway through JAKs, STAT3 can also be phosphorylated by some receptor tyrosine kinase (RTKs) and other non-receptor kinases (e.g., Src and Abl) [42]. Significantly, RTKs can directly activate STAT3 by independently of JAKs, and can also phosphorylate STAT3 by constitutively active non-receptor kinases [82]. In addition to tyrosine 705 phosphorylation, which is considered as a critical role in STAT3 activation, STAT3 is also active via serine (Ser727) phosphorylation by the MAPK or mTOR pathways [83, 84]. Moreover, recent evidence has identified that the reversible acetylation of STAT3 by histone deacetylases on lysine residue 685 provides a novel alternative mechanism of STAT3 activation [85].

### STAT3 inhibition

Negative regulators of STAT3 pathway, involving protein tyrosine phosphatases (PTPs), protein inhibitors of activated STAT (PIAS), and suppressor of cytokine signaling 3 (SOCS3), can inhibit STAT3 activity. CD45, a transmembrane PTP, inhibit STAT3 activity via dephosphorylation of JAKs [86]. PTP-1B, a non-transmembrane PTP, down-regulate leptin/STAT3 signaling via indirectly inactivating JAK2, which may be a new target for the treatment of obesity induced by leptin resistance [87]. SH2-containing protein tyrosine phosphatase (SHP-1 and SHP-2) can dephosphorylate and inactivate STAT3 by binding to the phosphorylation site of STAT3 [88]. PTP receptor T (PTPRT) specifically dephosphorylates STAT3 at Tyr705, thereby regulating cellular localization and expression of target genes [89]. In short, STAT3 activation is regulated by PTPs through direct and indirect dephosphorylation of p-STAT3. Moreover, PIAS3 binds to the STAT3 DNA binding domain and blocks the STAT3 transcriptional activity [90]. Additionally, SOCS3 act as negative feedback regulator of the JAK2/STAT3 signaling pathway by blocking STAT3 activation. The mechanisms involved in JAK2/STAT3 signaling inhibited by SOCS3 have been suggested, such as kinase-mediated inhibition of JAKs via the kinase inhibitory region (KIR) domain, binding site competitors of STAT3 for initiating JAKs, or degradation via the SOCS box [91, 92].

Another negative regulator Forkhead box O1 (FoxO1) is upregulated during early stages of diet-induced leptin resistance. Mice with ablation of FoxO1 in the POMC neurons are resistant to diet-induced obesity [93]. Our previous research demonstrated that FoxO1 inhibits STAT3 activation by preventing STAT3 from binding to specificity protein 1 (SP1)-POMC promoter complex, hence reducing POMC expression [94]. Various lines of evidence have suggested that direct FoxO1–STAT3 interaction is necessary for FoxO1 inhibition of POMC promoter activation.

## Leptin resistance

Leptin resistance defined by a failure response to high circulating leptin level, presenting a decreased capacity of leptin to suppress appetite or increase energy cost, leads to overweight, obesity and other metabolic disorders [95]. Leptin resistance is a main risk factor of obesity in humans [96]. To date, although much research work has been conducted in diet-induced obesity (DIO) micewith leptin resistance, the molecular mechanisms largely remains elusive. However, several theories have been put forth.

### Impairment in leptin transportation

**Fig. 4:**
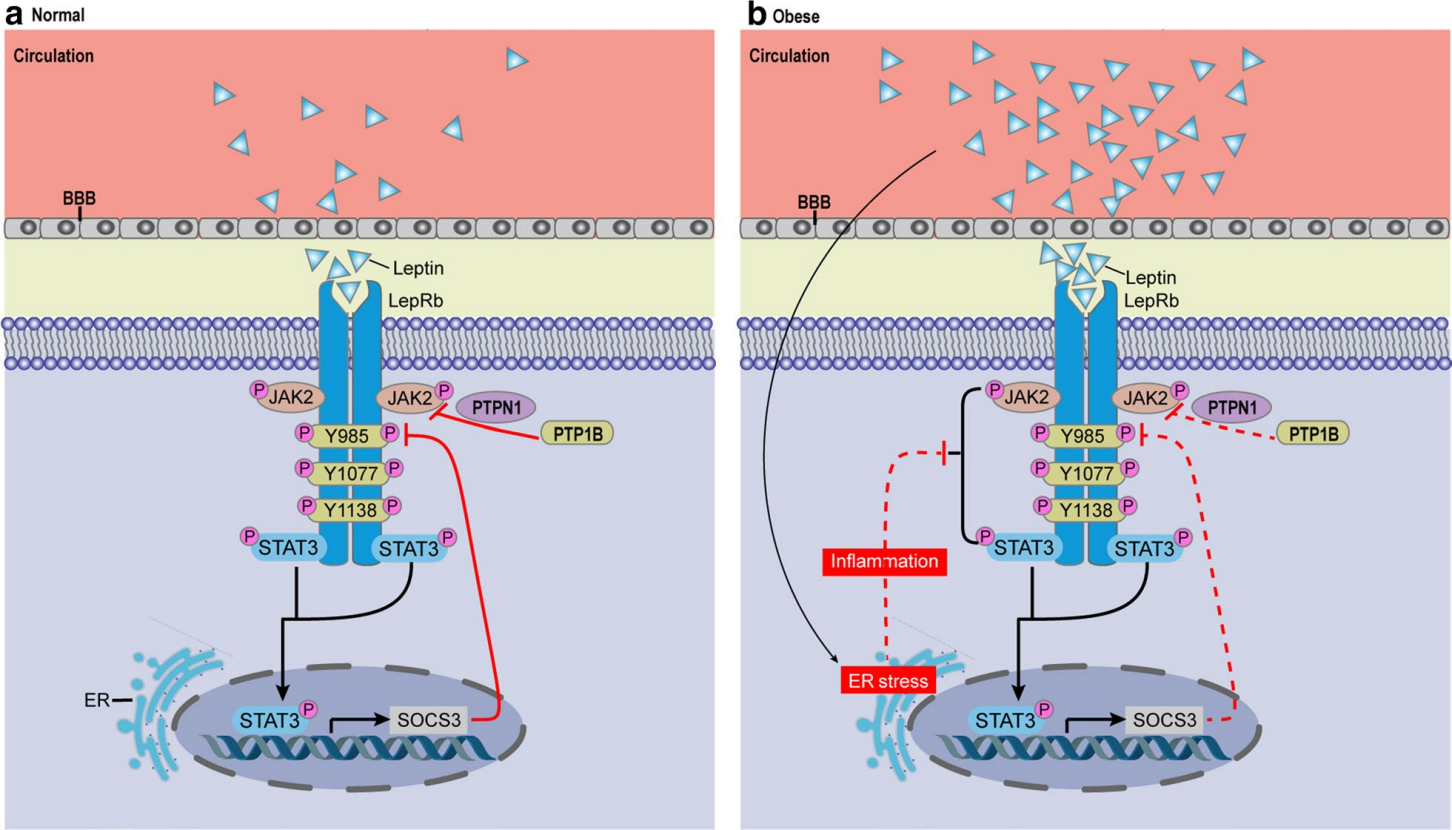
Mechanisms of Leptin resistance. **a** In animals with normal body weight, circulating leptin crosses the BBB and binds to LepRb, which induces phosphorylation of JAK2 and of multiple tyrosine residues in the LepRb intracellular domain. LepRb also receives inhibitory signals from multiple negative feedback loops, including SOCS3, PTP1B, ensuring that activation of LepRb does not go beyond a physiologically necessary point. **b** In obesity, circulating levels of leptin increase, which is associated with diminished leptin transport across the BBB and activation of the inhibitory negative feedback systems that eventually lead to diminished LepRb signaling. Many factors, including hyperleptinemia, inflammation, endoplasmic reticulum (ER) stress, inflammatory and defective autophagy, contribute to leptin resistance. ER stress and responses might contribute to a blunted physiological response to leptin in obesity

Reduced leptin transportation into the brain is considered as an important factor for leptin resistance [97]. To exert its actions, leptin needs to be transported into the brain across the blood–brain barrier (BBB) by a specific and saturable protein transporter [98] (Fig. 4). In this process, the LepRb is required and animal experiment shows the loss of LepRb reduces the quantity of leptin in the brain [99]. Hence, if the leptin level is excessively high, it may lead to LepRb saturation and further reduce the ratio of leptin transport via the BBB, finally causing leptin resistance [100] (Fig. 4b). Interestingly, several studies found the loss of leptin transporters do not lead to the decrease in the amount of leptin crossing the BBB [101, 102], but the molecular mechanism involved is still poorly understood. However, it is debatable whether leptin resistance is unrelated to transportation, due to decrease in leptin transport in obese subjects via the BBB. In a recent study, no difference was shown in leptin accumulation between obese and lean mice in the brain by visualizing leptin transportation into the brain with 3D imaging from lightsheet fluorescence microscopy [103]. Additionally, several factors have been determined to affect leptin transport. For example, epinephrine can enhance the leptin transportation across the BBB [104], whereas lipopolysaccharide [105], obesity [106], triglycerides [107], and fasting [108] largely reduce the rate of transport.

### Attenuation of leptin signaling

Defect in any element of the LepRb signaling pathway is expected to contribute to leptin resistance [109]. The decreased hypothalamic LepRb number and certain mutations of the *LepRb* gene with the truncated intracellular domain of LepRb, attenuate LepRb-mediated leptin signaling [18]. The upregulation of the negative regulators may diminish the leptin signaling, which is involved in the pathogenesis of leptin resistance [110, 111]. Additionally, the feedback inhibition and downregulation or inhibition of positive regulators of leptin signaling may also lead to leptin resistance [112–114].

### Deficiency in autophagy

Increasing evidence suggest the leptin-autophagy interaction plays a crucial role in the regulation of energy homeostasis. Ablation of autophagy-related protein 7 (Atg7) in the hypothalamus shows increased food intake and body weight, obesity and leptin resistance [115]. Moreover, Atg7-knockout mice exhibit leptin resistance due to the failure of leptin to activate STAT3 [116]. In contrast, mice with specific deletion of Atg7 in AgRP neurons showed reduced food intake and body fat, as well as a long term effect on energy homeostasis, suggesting improvement in leptin sensitivity [117]. Notably, a recent study indicated that leptin may contribute to the dysregulated/activated adipocyte autophagy and to its dysfunction in obesity [118].

### Endoplasmic reticulum stress and Inflammation

Endoplasmic reticulum (ER) stress and inflammation could impair leptin responsiveness of neuronal cells by blocking leptin signaling (Fig. 4). Recently, ER stress and the unfolded protein response (UPR) have emerged as a crucial link in the development of leptin resistance [119–121]. In particular, obese mice present ER stress in POMC neurons and peripheral tissues, suggesting that ER stress is induced by metabolic disorders related with obesity and HFD [122–124]. Notably, IKKβ/NF-κB is activated by HFD through elevated ER stress in the hypothalamus, which leads to leptin resistance [125]. Moreover, IKKβ expression enhances SOCS3 promoter activity, and this process is controlled by NF-κB. High fat diet (HFD) significantly induces SOCS3 in hypothalamus [125].

Induction of ER stress or deletion of the X-box binding protein 1 (Xbp1), a UPR transcription factor, results in leptin resistance and promotes weight gain on a HFD [119]. In contrast, the constitutive activation of Xbp1 in POMC neurons prevents against DIO and enhances leptin sensitivity [126]. Furthermore, ER stress inhibits leptin signaling in the hypothalamus via classical inhibitors of cytokine signaling (i.e. SOCS3 and PTP1B) [16, 127, 128] (Fig. 4). In line with this, ER stress can impair leptin signaling by blocking leptin-induced STAT3 phosphorylation, which is increasingly considered to be associated with leptin resistance [119, 120, 129]. It is noteworthy that a recent research demonstrated that leptin deficiency can cause elevated ER stress, while relieving ER stress can ameliorate metabolic regulation [130].

### Leptin-induced leptin resistance

Leptin itself is also a contributing factor in the development of leptin resistance, with this state termed “leptin-induced leptin resistance” [131]. The chronically augmented leptin in the CNS contributes to leptin resistance, which further promotes obesity, resulting in a vicious cycle of escalating metabolic disorder. Long-term exposure to hyperleptinemia causes leptin resistance by downregulating cellular response to leptin [132]. In contrast, low levels of plasma leptin highly ameliorates leptin sensitivity, yet does not prevent DIO [133]. Leptin sensitivity can be restored in mice by inhibiting leptin production in adipocyte and increasing leptin clearance in kidney [134]. Of note, triglycerides plays vital role in the leptin resistance to peripheral leptin on account of its inhibition of leptin transport across the BBB [97].

### Others

Apart from above-mentioned mechanisms, other factors also contribute to the leptin resistance. C-reactive protein (CRP) can increase BBB permeability, but augmented CRP may promote leptin resistance by blocking the binding of leptin to correspondent receptor [135]. Other potential mechanisms behind leptin resistance may be related to epigenetic remodeling [136]. More in-depth studies are required to further elucidate the mechanisms of leptin resistance.

## STAT3 phosphorylation in hypothalamus of DIO mice

STAT3 is specifically activated by leptin in vivo and in vitro. But how STAT3 regulates leptin’s biological activity has not been determined. Genetic STAT3 null or STAT3 phosphorylation deficiency in hypothalamus causes central leptin resistant and severe obesity animal [27, 137]. Leptin-activated STAT3 phosphorylation, as proved in the hypothalamus of lean wild-type animal [138], becomes a marker frequently employed in the assessment of central leptin signaling in the case of leptin resistance [15], similar to the situation in most obesity of humans and rodents of DIO [109]. Although a large portion of the mechanisms of leptin resistance remains to be identified, it raises a simple and salient question on whether the leptin-stimulated STAT3 activity is abated in the hypothalamus in DIO mice.

Most scientists claim that pSTAT3 induced by exogenous leptin treatment are reduced in the hypothalamus of DIO mice [139] evidenced mainly by the results from three different kind of investigations, and believe this decrease contributes to the mechanism of central leptin resistance [72, 140–146]. While immunohistochemical stain of STAT3 detects the local STAT3 distribution, arcuate nucleus (ARC) of hypothalamus is considered as the main leptin target for energy regulation [147], in spite of hypothesis that the anorectic effects of leptin are not specific from a brain region [148, 149]. In the majority of experiments using p-STAT3 immunohistochemistry, exogenous leptin-induced p-STAT3 increases in the ARC of lean mice, but not in that of DIO mice [109]; Western blot analysis is another method commonly employed in the detection of hypothalamic p-STAT3. The result of immunoblotting of hypothalamic arcuate nuclei lysate with the antibody against p-STAT3 demonstrates that leptin-activated STAT3 phosphorylation is blunted by 60% in the hypothalamus of leptin-resistant mice prepared by palmitate treatment [141]. The third approach for this purpose is eletrophoretic mobility shift assay (EMSA), which is a method to detect the DNA-binding activity of a certain protein. With DNA probe M67-SIE (cis inducible element) STAT3 activation is detected [26] in hypothalamus of lean mice, but not in that of DIO mice in response to exogenous leptin administration [145]. However, our previous work demonstrated that p-STAT3 induced by exogenous leptin treatment are increased in the hypothalamus of DIO mice, which may contributes to the mechanism of central leptin resistance [71].

Thus, if the onset and/or development of central leptin resistance results from the impairment of hypothalamic STAT3 activation, it is conceivable that the enhancement of STAT3 activity would somehow ameliorate this disorder. Whereas Ernst et al. reports the contrary result that high level of activated STAT3 in hypothalamus of the transgenic mice results in leptin resistance [133], it remains necessary to reevaluate the degree of STAT3 activation in hypothalamus of DIO mice in response to leptin administration and its effect on subsequential events such as POMC expression. Inconsistent to the prevailing consensus, Martin et al. showed that basal STAT3 phosphorylation is significantly elevated in arcuate of DIO mice [150]. In our previous report, we provided novel evidences from EMSA that both basal and exogenously leptin-mediated STAT3 activity was significantly elevated in the hypothalamus of DIO mice accompanied with decreased POMC expression and abnormal behaviors of metabolic physiology, suggesting that enhanced STAT3 activity negatively regulated leptin-mediated POMC expression in DIO mice. Furthermore, reduction of p-STAT3 by manual intervention furthered the leptin-mediated expression of POMC gene in the cultured cells, in line with the idea that p-STAT3 plays a negative role in hypothalamus of DIO mice [71]. Futhermore, evidences exist that excess STAT3 activity negatively regulated POMC expression in hypothalamus of DIO mice, suggesting the attenuation of STAT3 activity may facilitate leptin signaling [71]. Interestingly, over-expression of constitutively-active STAT3 in LepRb neurons leads to negative energy balance in ob/ob and wild-type mice, but not in DIO mice [34].

## Perspective

Central leptin resistance is one of the major problems in obese people. Currently investigation into the mechanism leads to diverse conclusion in terms of whether leptin contributes to the sensing of overfeeding-made fat expansion [151, 152]. Considering the essential role of the leptin axis to energy homeostasis in animals and humans has been convincingly established, we believe that a better understanding of the mechanism of signaling impairment is warranted. While basic question like the rising or descending of phosphor-STAT3 in hypothalamus of DIO mice is being debated, possibility still remains that leptin axis is able to sense overfeeding fat expansion. We previously proved that both basal and exogenously leptin-mediated STAT3 activity was significantly elevated in the hypothalamus of DIO mice, and speculated that leptin resistance happens downstream of STAT3 activation in the JAK2-STAT3 pathway of leptin signaling. Therefore, fully understanding of the detailed action of phosphor-STAT3 in hypothalamus will be of great significance. We propose that some unknown factor(s), which are abundant in hypothalamus of normal mice but gradually missed along the development of diet-induced obesity, would be necessary for phosphor-STAT3 to act on its target genes. The identification of these factors and the experimental evidences of direct interaction between phosphor-STAT3 and its target gene would hopefully illuminate our way to overcome leptin resistance and metabolic disorders.

## Data Availability

Not applicable.

## Abbreviations

STAT3: Signal transducer and activator of transcription 3
SHP2: SH2-containing protein tyrosine phosphatase 2
SOCS3: Suppressor of cytokine signaling 3
PTP1B: Protein tyrosine phosphatase 1B
DIO: Diet-induce obesity
PI3K: Phosphatidylinositol 3 kinase
MAPK: Mitogen-activated protein kinase
FoxO1: Forkhead box O1
IRS: Insulin receptor substrate
Grb2: Growth factor receptor-bound protein 2
CART: Cocaine- and amphetamine-regulated transcript
POMC: Proopiomelanocortin
AgRP: Agouti-related protein
NPY: Neuropeptide Y
VMH: Entromedial hypothalamus
NTS: Nucleus tractus solitaries
SF-1: Transcription factor steroidogenic factor 1
